# The Internet-Based Conversational Engagement Trial (I-CONECT): Theoretical Framework and Latest Findings.

**DOI:** 10.1093/geroni/igaf122.907

**Published:** 2025-12-31

**Authors:** Hiroko Dodge, Chao-Yi Wu, Liu Chen, Kexin Yu

**Affiliations:** Massachusetts General Hospital, Harvard Medical School, Boston, Massachusetts, United States; Massachusetts General Hospital, Harvard Medical School, Boston, Massachusetts, United States; Massachusetts General Hospital, Harvard Medical School, Boston, Massachusetts, United States; UT Southwestern Medical Center, Dallas, Texas, United States

## Abstract

Social isolation is a risk factor for dementia. In the recently completed randomized controlled trial, I-CONECT (www.i-conect.org; ClinicalTrials.gov: NCT02871921), we investigated the impact of frequent conversational interactions—a key component of social interactions—on cognitive functions. We hypothesized that engaging in frequent conversations enhances compensatory neural activity and helps maintain cognitive function, a concept framed by Park et al. (2013) as “scaffolding,” similar to Stern’s (2012) cognitive reserve theory. To specifically target the benefits of social bridging (Perry, 2012) separate from social bonding, we rotated interviewers each week. Participants in the experimental group engaged in semi-structured conversations with trained interviewers, prompted by daily themes and pictures, four times a week (30 minutes/session) for 6 months using user-friendly video-chat devices. The control group received brief (∼10-minute) weekly phone check-ins. A total of 186 socially isolated older adults aged 75 and older (86 with normal cognition, 100 with mild cognitive impairment [MCI]) were randomized. We previously reported that global cognitive function (the primary outcome), measured by the Montreal Cognitive Assessment (MoCA), improved by nearly 2 points in the MCI experimental group compared to the control group at 6 months (effect size: Cohen’s d = 0.73) (Dodge, 2024, doi: 10.1093/geront/gnad147). Functional MRI data suggested a trend toward increased connectivity in the dorsal attention network favoring the experimental group. Social satisfaction improved in both groups. During the symposium, we will explain the theoretical framework, recruitment, intervention approach, and findings to date, which can serve as a foundation for future behavioral trials of this kind.

